# Patients having surgery for ulnar nerve compression at the elbow rarely have affection of the spinal nerve root at C8-Th1 levels

**DOI:** 10.3389/fsurg.2022.1049081

**Published:** 2022-12-12

**Authors:** Erika Nyman, Alice Giöstad, Kasim Abul-Kasim, Lars B. Dahlin

**Affiliations:** ^1^ Department of Biomedical and Clinical Sciences, Linköping University, Linköping, Sweden; ^2^ Department of Hand Surgery, Plastic Surgery and Burns, Linköping University Hospital, Linköping, Sweden; ^3^ Primary Health Care Center Kolmården, Kolmården, Sweden; ^4^ Department of Radiology, Skåne University Hospital, Malmö, Sweden; ^5^ Department of Hand Surgery, Skåne University Hospital, Malmö, Sweden; ^6^ Department of Translational Medicine – Hand Surgery, Lund University, Malmö, Sweden

**Keywords:** ulnar nerve compression, cubital tunnel syndrome, spinal nerve roots, spinal cord, magnetic resonance imaging, intervertebral disc degeneration

## Abstract

Cervical pathology may contribute to residual problems after surgery for ulnar nerve compression. We aimed to evaluate the presence of pathological conditions in spinal cord and cervical spinal nerve roots in patients surgically treated for ulnar nerve compression at elbow. In a cohort of patients, surgically treated for ulnar nerve compression at elbow, magnetic resonance images (MRI; performed 3 years pre/postoperatively) were evaluated by a neuroradiologist blinded to patient characteristics and outcome of surgery. Cervical conditions were assessed and related to patient characteristics, preoperative McGowan grade, and outcome. Among 62 patients (45 unilaterally and 17 bilaterally), only one had spinal nerve root affection of nerve roots contributing to the ulnar nerve (C8-Th1). About half of the patients, mainly those at higher age, had alterations affecting C3–C7 spinal nerve roots at both surgically treated and contralateral, non-surgically treated, sides. Only few other changes were observed at cervical levels. A high McGowan grading was related to a high frequency of spinal nerve root affection. Smokers were more frequently observed among those with spinal nerve root affection at C3–C7 levels at surgically treated side. Residual problems, expressed as patient dissatisfaction and DASH score ≥40, were common. Spinal nerve roots, contributing to the ulnar nerve, are rarely affected in surgically treated patients with ulnar nerve compression at elbow even though pathology is often observed at other cervical levels. Pathology is often detected at other cervical spinal nerve root levels at surgically treated and contralateral sides, particularly among older patients, smokers, and in conjunction with worse preoperative McGowan grade. No relation between cervical pathology and outcome of ulnar nerve surgery is seen.

## Introduction

The ulnar nerve, originating from C8-Th1 spinal nerve roots, can be compressed at the elbow. Cervical radiculopathy, or other proximal lesions, may either produce similar or additional symptoms to an ulnar nerve compression, applying to “double crush syndrome” (1–3). The concept implies that neurons, compressed at one anatomical level, are more susceptible to an additional trauma at another level (4). An underlying neuropathy may also infer on neurons an increased vulnerability to compression (1, 5). A nerve compression is complex to treat when cervical spine and/or nerve root conditions are present (6–8). Outcome of surgery for nerve compression disorders with concomitant cervical pathology is poorer despite cervical spine surgery (8).

Evaluation of outcome after a surgically treated ulnar nerve compression is intricate, since several factors impact results (9, 10). Management of ulnar nerve compression must consider other potential proximal etiologies, with or without neck pain (11). Any association between nerve compression and coexisting cervical root lesions (12, 13) may not be related to the specific compressed nerve (2, 7). The variability of natural history of cervical spondylotic myelopathy, which may affect treatment decisions mainly for cervical problems and also relevant for symptomatology in ulnar nerve compression (14), should be considered. Imaging of spinal nerve roots and spinal cord with Magnetic Resonance Imaging (MRI) may reveal detailed pathology (15–18). Our aims were to blinded, and in detail, evaluate cervical pathology at all cervical levels from pre- and postoperatively obtained images of MRIs and relate findings to preoperative symptoms as well as outcome of surgery for ulnar nerve compression at the elbow from part of an earlier described patient cohort (19).

## Materials and methods

### Patient characteristics

**Figure 1:**
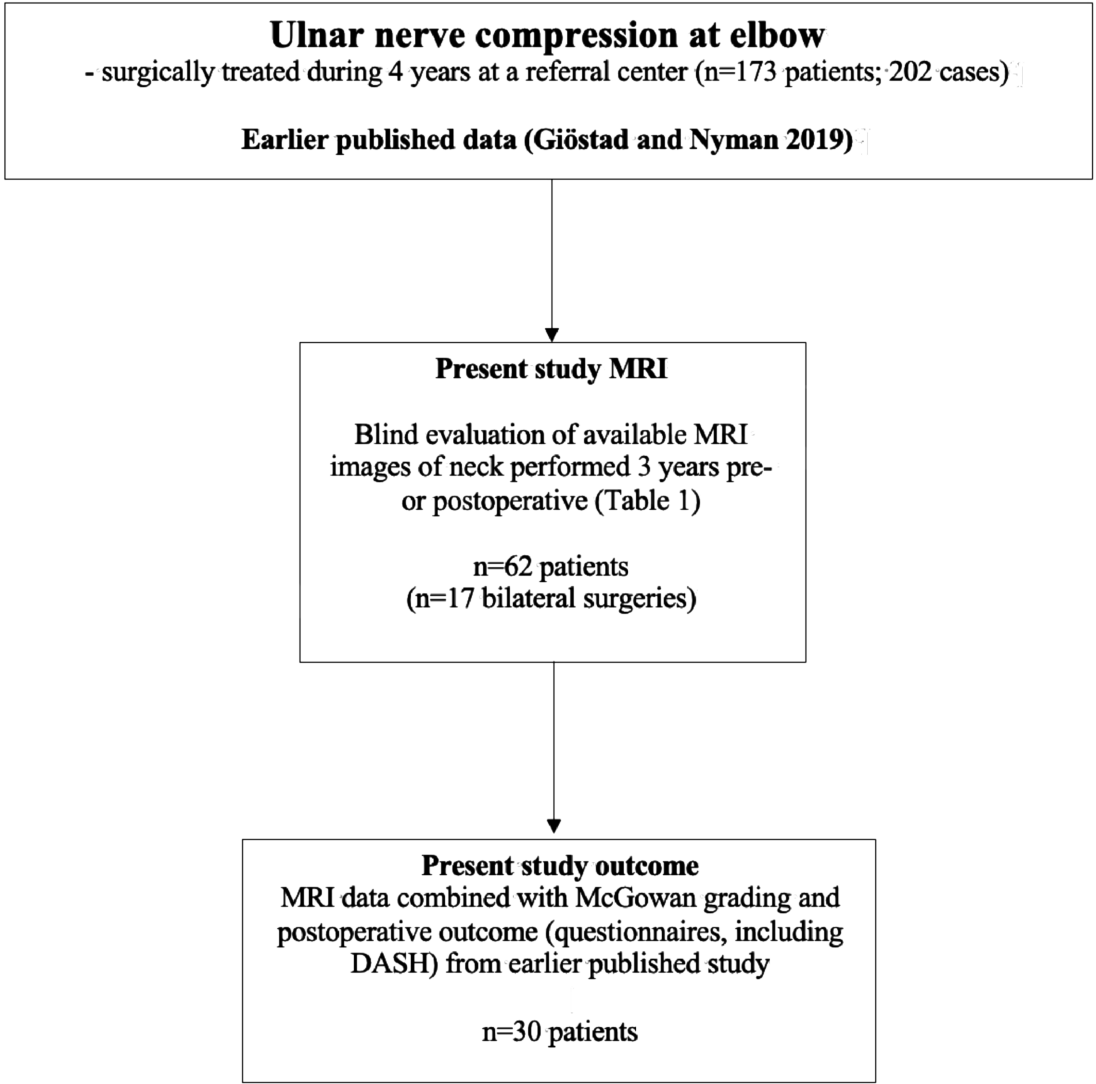
Flow chart of included patients in relation to previously published study of surgically treated patients (19). For variables, please see Table 1.

A retrospective observational cohort study of ulnar nerve compression at the elbow at Department of Hand Surgery, Plastic Surgery and Burns, Linköping University Hospital, Linköping, Sweden, where outcome of surgery was analyzed in 173 patients [29 bilateral surgery; thus, in total 202 cases (19)], was earlier performed. A majority of the original 173 patients (202 cases) had a concomitant affection of another nerve in operated arm and one third had a concomitant affection of another nerve in contralateral arm (Figure 1). Around 50% had an MRI performed preoperatively, with referral to radiology of whom 30% had signs of nerve root affection (level not specified) according to the patients' charts. For details of the patient cohort see Giöstad and Nyman 2019 (19).

**Table 1:** Definition of assessment of the MRI images in 62 patients with surgically treated ulnar nerve compression at the elbow.

*General evaluation:*
Disc herniation (location, side, level)
Cervical spinal stenosis (central)
Spinal nerve root affection (side, level) judged as:
Normal
Probable
Definite
Spinal cord compression
Increased T2 signal in spinal cord
Disc degeneration (level)
Modic I (Modic type I endplate change)
*Specific evaluation at C3-Th1 levels uni- and bilaterally:*
Spinal nerve root affection
No affection of spinal nerve roots
Probable involvement of spinal nerve roots^a^
Definite involvement of spinal nerve roots^a^

^a^ Initially spinal nerve root affection was divided in no, probable and definite involvement, but in the statistical analyzes probable and definite are merged into the two categories “no spinal nerve root affection” or “spinal nerve root affection”. For details see Methods.

In the present study, pre- or postoperative available MRI images were retrieved and re-evaluated (for details see below). The inclusion criterion of the present study was availability of MRI examination with images that could be re-evaluated as described below. Background data of the present 62, out of the earlier presented 173, patients were extracted from the file based on the patient charts, including age, sex, uni- or bilateral surgery, McGowan grading for severity of preoperative ulnar nerve problems (20), other nerve compression lesion(s) and surgery for other hand condition in the same or opposite arm, shoulder or neck problems, and presence of polyneuropathy and diabetes. Data of outcome was also extracted from the file, consisting of DASHscore and replies from two further questions: “How do you think the hand/works today compared to before surgery?” and “Are you pleased with the result from the surgery?”; both graded as completely fine – improved vs. unchanged—worse; data from 173 patients earlier published (19).

### Evaluation of MRI images

**Figure 2:**
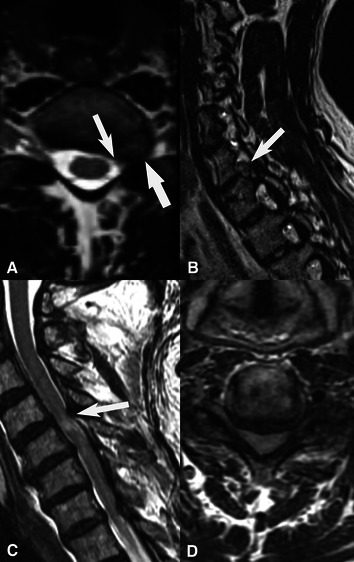
MRI of two different patients that had a history of ulnar nerve compression with a diagnosis resulting in surgical treatment of the nerve compression disorder at the elbow, with and without another nerve compression disorder. (**A,B**) T2W axial and sagittal images showing left sided foraminal disc herniation C7-Th1 (thick arrow) with compression of the C8-nerve root (thin arrow, A). (**C,D**) T2W sagittal and axial images of another patient with severe central stenosis with total obliteration of CSF spaces around the spinal cord (**D**) and signal changes in the spinal cord at level C4-C5 (thin arrow C).

**Figure 3:**
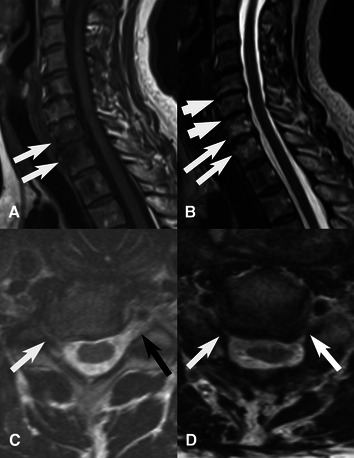
MRI of three different patients. (**A,B**) T1W-, and T2W sagittal images show Modic type 1 changes with bone marrow oedema around C7-Th1-disc space (thin long arrows) and moderate disk degeneration C5–C6 and C6–C7 (thick short arrows). (**C**) T2W axial image shows right sided foraminal stenosis C6–C7 with severe narrowing of neural foramen compression (white arrow) and C7-nerve root. Black arrow shows normal left neural foramen. (**D**) T2W axial image shows bilateral foraminal stenosis at C5–6 level with C6-nerve root compression.

MRI images from the present 62 patients (Figure 1), investigated within the last 3 years before or after surgery, were available for re-evaluation and re-analyzed by a single experienced neuroradiologist (KAK), who was not aware of any clinical patient data, including affected side, if surgery was performed bilaterally, presence of any concomitant nerve compression disorder or outcome of surgery. The evaluation included assessment of presence of disc herniation (location, side, level), cervical spinal (central) stenosis, any spinal nerve root affection (side and level; graded as normal, probable affection and definite affection), spinal cord compression, increased T2 signal in spinal cord, disc degeneration (level) and Modic I (Modic type I endplate change) (Figures 2, 3). The Modic type endplate changes represent a classification for vertebral body endplate MRI signal. Modic type I is defined as T1: low signal; T2: high signal; represents bone marrow oedema and inflammation (Table 1). Spinal nerve root affection was defined as (a) no affection, (b) a probable involvement of the specific nerve root and (c) a definite involvement of a specific nerve root at the individual C3-Th1 nerve root levels bilaterally. Data were then further categorized into (a) no spinal nerve root affection, or (b) spinal nerve root affection C8–Th1 (origin of the ulnar nerve), and spinal nerve root affection of C3–C7, respectively, based on overall judgment, where a probable involvement was defined as a definite affection if a disc herniation, a central stenosis or a disc degeneration were present (Table 1).

### Statistical analyses

Data are presented as numbers (*n*; %) or median (interquartile range; IQR). Any differences in categorial data were analyzed with *χ*^2^-test or Fisher’s exact test and in continuous data (not-normally distributed) with the Mann-Whitney *U* Test. The significance level was set at a *p*-value of <0.05. Statistical analyses were performed with IBM SPSS Statistics (version 26; 2019).

All methods were performed in accordance with relevant guidelines and regulations, including the Declaration of Helsinki.

## Results

### Characteristics of the patients

Among 62 patients, 39 were women and 23 were men [latter statistical higher age (*p *= 0.012) and more frequently observed polyneuropathy (*p* = 0.016); no other significant differences were seen between sex] (Table 2). Among all patients (*n* = 61; information missing in one case), 15 (25%) were smokers. Smokers were more frequently observed among cases with spinal nerve root affection at C3–C7 levels at surgically treated side (*p* = 0.039; Table 3), but without any other significant differences in smoking habits. Unilateral surgery was performed in 45 patients (right side 23 patients; left side 22 patients) and bilateral surgery in 17 patients with a similar distribution of McGowan grading 1–3 (Table 2). Another compression neuropathy was frequently present in the same (39/62 patients; 63%) or contralateral (29/62; 47%) arm. A hand surgical co-morbidity, treated at the same time as ulnar nerve compression, was observed in 24/62 (39%) patients. Shoulder problems were less frequently reported (11/62; 18%). Neck problems were observed in around half of the patients (29/62; 47%). Data are summarized in Tables 2, 3.

**Table 2:** Characteristics of patients with surgically treated ulnar nerve compression at the elbow and with available MRI of neck.

	All patients (*n* = 62)	Women (*n* = 39)	Men (*n* = 23)
Age (years)	46 [40–58]	43 [34–51]	55 [42–63]
Sex (women/men)	39/23 (63/37)	NA	NA
Smokers^a^	15 (25)	10 (26)	5 (22)
Surgery (unilateral/bilateral)	45/17 (73/27)	29/10 (74/26)	16/7 (70/30)
McGowan grading (grade 1/2/3) in 62 patients^b^	21/18/23 (34/29/37)	12/15/12 (31/38/31)	9/3/11 (39/13/48)
McGowan grading (grade 1/2/3) in 17 patients^c^	7/5/5 (41/29/29)	4/4/2 (40/40/20)	3/1/3 (43/14/43)
Other neuropathy – surgically treated side	39 (63)	27 (69)	12 (52)
Other neuropathy – contralateral side	29 (47)	17 (44)	12 (52)
Co-morbidity – surgically treated side	24 (39)	16 (41)	8 (35)
Shoulder problems	11 (18)	7 (18)	4 (17)
Neck problems	29 (47)	18 (46)	11 (48)
Polyneuropathy	4 (6)	0 (0)	4 (17)
Diabetes^d^	9 (15)	4 (10)	5 (23)

Characteristics of 62 patients surgically treated for ulnar nerve compression at the elbow and with available preoperative or postoperative MRI of neck. MRI examinations performed pre- or postoperative (±3 years in relation to surgery).

Data presented as *n* (%) or median [interquartile range, IQR].

^a^ Missing data in one case.

^b^ McGowan grading on surgically treated side (unilateral *n* = 45 and first surgical side of patients with bilateral diagnosis *n* = 17; thus *n* = 62).

^c^ McGowan grading on second surgical side in bilaterally treated cases (*n* = 17).

^d^ Missing data in two cases.

**Table 3:** Cervical spinal nerve root affection at surgically treated side and at any side of patients with ulnar nerve compression at the elbow and with available MRI.

	No spinal nerve affection at C3–C7 levels at surgically treated side^a^ (*n* = 36)	Spinal nerve affection at C3–C7 levels at surgically treated side^a^ (*n* = 26)	No spinal nerve affection at C3-Th1 levels at any side (*n* = 31)	Spinal nerve affection at C3-Th1 levels at any side (*n* = 31)
Age (years)	43 [34–48]	57 [43–64]	42 [33–47]	55 [42–63]
Sex (women/men)	26/10 (72/28)	13/13 (50/50)	22/9 (71/29)	17/14 (55/45)
Smokers^a^	5 (14)	10 (38)	4 (13)	11 (35)
Surgery (unilateral/bilateral)	25/11 (69/31)	20/6 (77/23)	20/11 (65/35)	25/6 (81/19)
McGowan grading (grade 1/2/3) in 62 patients^b^	15/13/8 (42/36/22)	6/5/15 (23/19/58)	14/11/6 (45/36/19)	7/7/17 (23/23/55)
McGowan grading (grade 1/2/3) in 17 patients^c^	6/3/2 (55/27/18)	1/2/3 (17/33/50)	6/3/2 (55/27/18)	1/2/3 (17/33/50)
Other neuropathy – surgically treated side	25 (69)	14 (54)	22 (71)	17 (55)
Other neuropathy – contralateral side	16 (44)	13 (50)	16 (52)	13 (42)
Co-morbidity – surgically treated side	15 (42)	9 (35)	11 (35)	13 (42)
Shoulder problems	5 (14)	6 (23)	5 (16)	6 (19)
Neck problems	15 (42)	14 (54)	14 (45)	15 (48)
Polyneuropathy	0 (0)	4 (15)	0 (0)	4 (13)
Diabetes^d^	4 (11)	5 (21)	3 (10)	6 (21)

Characteristics of 62 patients surgically treated for ulnar nerve compression at the elbow and with available preoperative or postoperative MRI of neck. MRI examinations performed pre- or postoperative (±3 years in relation to surgery).

Data presented as *n* (%) or median [interquartile range, IQR].

^a^ Indicates that single patient with affection of the Th1 spinal nerve root is also included since the patient had spinal nerve root affection similarly at C3–C7 levels. ^a^Missing data in one case. ^b^McGowan grading on surgically treated side (unilateral *n* = 45 and first surgical side of patients with bilateral diagnosis *n* = 17; thus *n* = 62). ^c^McGowan grading on second surgical side in bilaterally treated cases (*n* = 17). ^d^Missing data in two cases.

### General observation of cervical pathology

In three patients, a single spinal nerve root at C3–C7 level was judged as a probable affection without any other pathology and in one patient a bilateral probable affection was observed at C7 level. These changes were also defined as spinal nerve root affection. Most changes were observed in C3–C7 spinal nerve roots. Affection of spinal nerve roots C3–C7 at surgically treated side was seen in almost half of the cases (26/62; 42%), while C8-Th1 spinal nerve root affection was rarely observed (1/62; 2%; Table 4, Supplementary Table S1). The patient with C8-Th1 spinal nerve root affection at the surgically treated side (unilateral surgery) had also C3–C7 spinal nerve root affection at the ipsi- and contralateral sides. Spinal nerve root affection at C3–C7 levels at contralateral side was seen in about the same frequency as for surgically treated side (39% and 42%, respectively) among all patients. The single case with C8-Th1 involvement had affection at the surgically treated side (Table 4). Fifty percent (31/62) of the patients had nerve root affection at any side or level of the cervical spinal nerve roots (Table 4). Disc herniation, cervical spinal stenosis, medulla compression, increased T2 signal in the spinal cord, Modic I or disc degeneration were less frequently observed (Supplementary Table S2).

**Table 4:** MRI findings in neck in surgically treated patients with ulnar nerve compression at the elbow.

	All patients (*n* = 62)	Women (*n* = 39)	Men (*n* = 23)
Spinal nerve root affection surgically treated side C3–C7	26 (42)	13 (33)	13 (56)
Spinal nerve root affection surgically treated side C8-Th1	1 (2)	0 (0)	1 (4)
Spinal nerve root affection contralateral side C3–C7	24 (39)	13 (33)	11 (48)
Spinal nerve root affection contralateral C8-Th1	0 (0)	0 (0)	0 (0)
Spinal nerve root affection C3-Th1 at any side	31 (50)	17 (44)	14 (61)

MRI findings in available MRI examinations in neck in available 62 patients surgically treated with decompression due to ulnar nerve compression at the elbow. MRI examinations performed pre- or postoperative (±3 years in relation to surgery). Spinal nerve root affection and disc herniation: probable (slight-moderate) or definite (severe).

Data presented as *n* (%).

There was a significant difference in age between the patients with (*n* = 31) and without (*n* = 31) spinal nerve root affection C3-Th1 at any side (*p* = 0.001), as well as those with (*n* = 26) and without (*n* = 36) spinal nerve root affection at C3–C7 at surgically treated side (*p* = 0.001), showing a higher age in those with spinal nerve root affection (Table 3). No differences regarding sex were observed between those with and without spinal nerve root affection (*p* > 0.05). Generally, men were significantly older than women among all patients (*p* = 0.012, Table 3). There were no differences concerning a concomitant neuropathy in the same or in the contralateral arm and spinal nerve root affection at surgically treated or contralateral side, respectively, at C3–C7 spinal nerve roots (*p* = 0.29 and *p* = 0.30, respectively) or C3-Th1 spinal nerve root affection at any side (*p* = 0.29 and *p* = 0.61, respectively, Table 3). There were no differences between unilateral and bilateral cases concerning presence of spinal nerve root affection at surgically treated or contralateral sides at C3–C7 spinal nerve roots (*p* = 0.58, and *p* = 0.78, respectively) or C3-Th1 spinal nerve roots at any side (*p* = 0.26; Table 3).

### McGowan grading and spinal nerve root affection

There were differences concerning C3–C7 spinal nerve root affection at surgically treated and contralateral sides in relation to McGowan grading among all cases (*p* = 0.017 and *p* = 0.022, respectively; Table 5) with McGowan grade 3 more frequently observed in those with nerve root affection. McGowan grade 3 was also related to more frequently observed ipsilateral, but not contralateral, pathology at C3–C7 spinal nerve roots among surgically treated patients at unilateral side (*p* = 0.045). Analyses of bilateral cases (*n* = 17 patients; *n* = 34 cases) showed no relation between spinal nerve root affection and McGowan grading (*p* = 0.06; Table 5).

**Table 5:** McGowan grading and spinal nerve root affection in patients with surgically treated ulnar nerve compression at the elbow.

	McGowan grade 1	McGowan grade 2	McGowan grade 3	*p*-value
Spinal nerve root affection at C3–C7 levels at surgically treated side *n* = 62 patients	6 (29) (*n* = 21)	5 (28) (*n* = 18)	15 (65) (*n* = 23)	**0**.**017**
Spinal nerve root affection at C3–C7 levels at contralateral side *n* = 62 patients	5 (24) (*n* = 21)	5 (28) (*n* = 18)	14 (61) (*n* = 23)	**0**.**022**
Spinal nerve root affection at C3–C7 levels at surgically treated side *n* = 45 unilateral patients	5 (36) (*n* = 14)	4 (27) (*n* = 15)	11 (69) (*n* = 16)	**0**.**045**
Spinal nerve root affection at C3–C7 levels at contralateral side *n* = 45 unilateral patients	4 (29) (*n* = 14)	4 (27) (*n* = 15)	10 (62) (*n* = 16)	0.073
Spinal nerve root affection at C3–C7 levels at surgically treated side^a^ *n* = 34 cases based on 17 bilateral patients	2 (14) (*n* = 14)	3 (38) (*n* = 8)	7 (58) (*n* = 12)	0.064

McGowan grading and presence of spinal nerve root affection at C3–C7 levels in available MRI examinations at surgically and contralateral sides in patients surgically treated for ulnar nerve compression at the elbow. MRI examinations performed pre-or postoperative (±3 years in relation to surgery).

Values are (*n*; %). *p-*values obtained from *χ*^2^-test. Significant *p*-values are marked in bold.

^a^ Spinal nerve root affection at C3–C7 levels at surgically treated side for 17 bilateral patients (34 cases) equal to spinal nerve root affection at any side.

### Relation between spinal nerve root affection at C3–C7 levels

There was a relation between presence of C3–C7 spinal nerve root affection at surgically treated and contralateral sides among patients with unilateral (*p* = 0.005) ulnar nerve compression at the elbow (Supplementary Table S3).

### Outcome in relation to sex and spinal nerve root affection

Residual problems were common [median DASH score 40 (10–51) at minimum follow up time 12 months; *n* = 30; no differences in sex; *p* = 0.80]. No significant differences in DASH scores between no affection and affection at surgically treated side of C3–C7 spinal nerve roots (*p* = 0.46) or no affection and affection of C3-Th1 spinal nerve roots at any side (*p* = 0.30) were detected. Generally, 53% of the patients (*n* = 30) expressed completely fine or improved hand function as result of surgery with respect to the two questions: “How do you think the hand/works today compared to before surgery?” (completely fine – improved vs. unchanged—worse) and “Are you pleased with the result from the surgery?” (completely fine – improved vs. unchanged - worse); no sex differences (*p* = 0.47 and *p* = 0.47, respectively). Concerning the two outcome questions, there were no relations between no affection and affection at surgically treated side of C3–C7 spinal nerve roots (*p* = 0.72 and *p* = 0.72, respectively) or no affection and affection of C3-Th1 spinal nerve roots at any side (*p* = 0.48 and *p* = 0.48, respectively). Data is shown in Supplementary Tables S4.

## Discussion

In patients with unilaterally or bilaterally surgically treated ulnar nerve compression at the elbow, spinal nerve root pathology was rarely seen in spinal nerve roots distributing nerve fibers to the ulnar nerve (C8-Th1). Pathology was seen in about half of the patients at other cervical spinal nerve root levels (C3–C7) at the same or contralateral sides as surgery was performed. These alterations with impact on cervical C3–C7 spinal nerve roots were more often observed in patients at higher age, among smokers and in patients with higher McGowan grade, but did not influence surgical outcome in accordance with some published studies using other evaluation methods (2, 7, 10, 12, 13).

We did not observe any impact of C8-Th1 spinal nerve root pathology on symptomatology in these patients with ulnar nerve compression. A concomitant nerve compression lesion, i.e., other neuropathy, on surgically treated or contralateral sides was present in up to 63% among all operated cases. No association between cervical spinal nerve root pathology and other ipsi- or contralateral nerve compression lesions was detected; thus not supporting the “double crush syndrome” concept (1). One may interpret the results as that the surgeons treating the present cases in some way excluded patients with any cervical pathology from surgery. On the other hand more symptoms and disability, graded according to McGowan (20), were associated with significantly more frequent C3–C7 spinal nerve root pathology both at surgically treated and contralateral sides. This indicates that cervical pathology may predispose for symptoms interpreted as a nerve compression lesion. Furthermore, one may suggest that another more precise grading system may be used for ulnar nerve compression at the elbow even if other systems also have been presented (21, 22). A clinically applicable grading system of muscle function has been reported to differentiate ulnar nerve compression from a C8-Th1 radiculopathy (23), with data of ulnar and median nerve innervated forearm muscles from the C8 and Th1 spinal nerve roots presented (24).

Spinal nerve root pathology was more frequently noted in patients at higher age, indicating age as an important factor for cervical pathology (17, 25), particularly observed as disc degeneration in asymptomatic patients over 60 years of age (15, 16). The patients that smoked had more often C3–C7 spinal nerve root affection, which may be related to characteristics of patients undergoing surgery for ulnar nerve compression and not presence or progression of cervical pathology (26, 27). Degenerative changes, often at C5–C6 levels, detected by MRI are reported to be around 60% and seem to commonly progress over time with an association between foraminal stenosis and onset of upper-limb pain (18) and with lumbar disc herniation (26). An epidemiological survey of cervical radiculopathy, with a cohort of similar age as the present study, showed that a monoradiculopathy most frequently affects particularly the C7, followed by the C6, nerve roots (Radhakrishnan et al.). Furthermore, the survey also showed a similar presence of disc protrusion and spondylosis, and during a median follow up of around 5 years the recurrence of the condition was reported to be high (31%) (28). Among our patients (median age 46 years), around 50% had spinal nerve root affection at any side being much higher than a similar volunteer age cohort (foraminal stenosis 10% and 20% at 40–49 and 50–59 years, respectively) (18). These data should be related to present findings of more commonly spinal nerve root affection in relation to more severe McGowan grading (20). Recently, age and cervical spinal disc herniation, the latter being 18% in the present study, were reported to be associated with increased risk of revision surgery for ulnar nerve compression at the elbow. In addition, sex, BMI, smoking and other comorbidities seemed not be related to such revision surgery (29). However, the anatomic levels of the spinal disc degeneration were not defined. Our study, and the mentioned epidemiological survey (28), indicates that pathology may be more relevant at other cervical levels than those related to the ulnar nerve.

Our patients experienced residual problems. Absence of relation between spinal nerve root affection and patient reports from DASH and the used two questions illustrate complexity of surgery for ulnar nerve compression at the elbow, considering cervical pathology.

A present limitation is our retrospective design with MRI investigations performed within the last three years before or after surgery. An optimal option would have been to perform an MRI at a specific time point in relation to the diagnosis and the surgical treatment of the nerve compression disorder. However, in this retrospective study we did not have that opportunity. Another limitation is the relatively small sample size resulting in a restriction to add additional statistical analyses, e.g., a logistic regression, and the possibility to relate to confounding factors. There is also a risk of selection bias in the present patient cohort, since patients with available MRI may be more likely to have cervical spine pathology compared to the whole group of original 173 patients. A strength is the neuroradiologist’s unawareness of any clinical data and the meticulous grading system with several variables. Nevertheless, outcome of surgery seems not to be directly influenced by cervical pathology; thus, ulnar nerve compression at the elbow being unpredictable to treat.

We conclude that spinal nerve roots at C8-Th1-levels, contributing to the ulnar nerve, are rarely affected in patients surgically treated for ulnar nerve compression at the elbow. Pathology is often observed at other cervical spinal nerve root levels at surgically treated and contralateral sides, particularly in older patients, among smokers, and in conjunction with worse preoperative McGowan grade. However, no relation between cervical pathology and outcome of ulnar nerve surgery was seen.

## Data Availability

The original contributions presented in the study are included in the article/supplementary materials, further inquiries can be directed to the corresponding author/s.
