# Supplementary material for: Patients having surgery for ulnar nerve compression at the elbow rarely have affection of the spinal nerve root at C8-Th1 levels
Source: Front Surg. 2022 Dec 12;9:1049081. doi: 10.3389/fsurg.2022.1049081 (PMC9790900; doi:10.3389/fsurg.2022.1049081)
Supplement: Supplementary file 1 [file Datasheet1.pdf]

Supplemental table S1 MRI findings concerning spinal nerve root affection in neck in surgically treated patients with ulnar nerve compression at the elbow.

	**All patients (n=62)**	**No spinal nerve affection at C3-C7 levels at surgically treated side* (n=36)**	**Spinal nerve affection at C3-C7 levels at surgically treated side* (n=26)**	**No spinal nerve affection at C3-Th1 levels at any side (n=31)**	**Spinal nerve affection at C3-Th1 levels at any side (n=31)**
Spinal nerve root affection surgically treated side C3-C7	26 (42)	NA	NA	0 (0)	26 (84)
Spinal nerve root affection surgically treated side C8-Th1	1 (2)	0 (0)	1 (4)	0 (0)	1 (3)
Spinal nerve root affection contralateral side C3-C7	24 (39)	5 (14)	19 (73)	0 (0)	24 (77)
Spinal nerve root affection contralateral side C8-Th1	0 (0)	0 (0)	0 (0)	0 (0)	0 (0)
Spinal nerve root affection C3-Th1 at any side	31 (50)	5 (14)	26 (100)	NA	NA

MRI findings in neck in available 62 patients surgically treated with decompression due to ulnar nerve compression at the elbow with available MRI examinations performed pre- or postoperative (± 3 years in relation to surgery). Data presented as n (%).

* Indicates that single patient with affection of the Th1 spinal nerve root is also included since the patient had spinal nerve root affection similarly at C3-C7 levels.

Supplemental table S2. MRI findings concerning other alterations than spinal nerve root affection in neck in surgically treated patients with ulnar nerve compression at the elbow.

	All patients (n=62)	Women (n=39)	Men (n=23)	No spinal nerve affection at C3-C7 levels at surgically treated side (n=36)	Spinal nerve affection at C3-C7 levels at surgically treated side (n=26)	No spinal nerve affection at C3-Th1 levels at any side (n=31)	Spinal nerve affection at C3-Th1 levels at any side (n=31)
Disk herniation	11 (18)	6 (15)	5 (22)	3 (8)	8 (31)	2 (6)	9 (29)
Cervical spinal stenosis[a]	2 (3)	1 (3)	1 (4)	0 (0)	2 (8)	0 (0)	2 (7)
Medulla compression	3 (5)	1 (3)	2 (9)	0 (0)	3 (11)	0 (0)	3 (10)
Increased T2-signal medulla	3 (5)	1 (3)	2 (9)	0 (0)	3 (11)	0 (0)	3 (10)
Modic I	3 (5)	3 (8)	0 (0)	1 (3)	2 (8)	0 (0)	3 (10)
Disc degeneration	22 (36)	12 (31)	10 (44)	5 (14)	17 (65)	2 (7)	20 (65)

MRI findings in neck in available 62 patients surgically treated with decompression due to ulnar nerve compression at the elbow with available MRI examinations performed pre- or postoperative (± 3 years in relation to surgery). Data presented as n (%).

[a]Missing data in one case.

Supplemental table S3. Unilateral surgery for ulnar nerve compression at the elbow related to affected nerve roots.

	Spinal nerve root affection at C3-C7 levels at contralateral side	No spinal nerve root affection at C3-C7 levels at contralateral side	p-value
**Unilateral ulnar nerve compression (n=45 patients)**			
Spinal nerve root affection at C3-C7 levels at surgically treated side	13 (29)	7 (16)	**0.005**
No spinal nerve root affection at C3-C7 levels at surgically treated side	5 (11)	20 (44)	

Unilateral surgery for ulnar nerve compression at the elbow in relation to affected nerve roots C3-C7 at contralateral side with available MRI examinations performed pre-or postoperative (± 3 years in relation to surgery).
Values are n (%). p-values obtained from Chi^2-test. Significant p-values are marked in bold.

Supplemental table S4. Outcome in relation to sex and affected spinal nerve roots in patients (n=30) with ulnar nerve compression at the elbow.

	**All patients (n=30)**	**Women (n=19)**	**Men (n=11)**	**No spinal nerve affection C3-C7 at surgically treated side* (n=15)**	**Spinal nerve affection C3-C7 at surgically treated side* (n=15)**	**No spinal nerve affection C3-Th1 at any side* (n=13)**	**Spinal nerve affection C3-Th1 at any side* (n=17)**
DASH at follow up (> 12 months)	40 [10-51]	41 [8-52]	28 [11-48]	38 [9-50]	43 [21-55]	27 [8-48]	43 [22-54]
How do you think the hand works today compared to before surgery? (completely fine-improved vs. unchanged-worse)	16/14 (53/47)	9/10 (47/53)	7/4 (64/36)	9/6 (60/40)	7/8 (47/53)	8/5 (62/38)	8/9 (47/53)
Are you pleased with the result from the surgery? (completely fine-improved vs. unchanged-worse)	16/14 (53/47)	9/10 (47/53)	7/4 (64/36)	9/6 (60/40)	7/8 (47/53)	8/5 (62/38)	8/9 (47/53)

Data based on DASH questionnaire and two other questions as indicated in table. Data from the two questions were pooled as “completely fine-improved” versus “unchanged-worse”. MRI examinations were performed pre-or postoperative (± 3 years in relation to surgery) and judgment was made on available MRI examinations.
* indicates that the single patient with affection of only the C8-Th1 spinal nerve root is excluded (no reply from questionnaire/questions).
Data are presented as median [interquartile range, IQR] or (n; %).
For p-values see Results.
